# 2014 Ebola Outbreak: Media Events Track Changes in Observed Reproductive Number

**DOI:** 10.1371/currents.outbreaks.e6659013c1d7f11bdab6a20705d1e865

**Published:** 2015-04-28

**Authors:** Maimuna S. Majumder, Sheryl Kluberg, Mauricio Santillana, Sumiko Mekaru, John S. Brownstein

**Affiliations:** Massachusetts Institute of Technology, Engineering Systems Division, Cambridge, Massachusetts, USA; Boston Children’s Hospital, Harvard Medical School, Boston, Massachusetts, USA; School of Engineering and Applied Sciences, Harvard University, Cambridge, Massachusetts, USA; Boston Children's Hospital, Emergency Medicine, Boston, Massachusetts, USA; Boston Children’s Hospital, Harvard Medical School, Boston, Massachusetts, USA

**Keywords:** ebola, interventions, Liberia, media, reproductive number, Sierra Leone

## Abstract

In this commentary, we consider the relationship between early outbreak changes in the observed reproductive number of Ebola in West Africa and various media reported interventions and aggravating events. We find that media reports of interventions that provided education, minimized contact, or strengthened healthcare were typically followed by sustained transmission reductions in both Sierra Leone and Liberia. Meanwhile, media reports of aggravating events generally preceded temporary transmission increases in both countries. Given these preliminary findings, we conclude that media reported events could potentially be incorporated into future epidemic modeling efforts to improve mid-outbreak case projections.

## Commentary

The ongoing Ebola epidemic in West Africa is significantly larger and more widespread than any other in history. While previous outbreaks in small villages have burned out due to the local depletion of susceptible individuals, this epidemic has spread across entire countries, and thus can only be curtailed by interventions aimed at reducing new infections across all locations.^1^ The efficacy of large-scale interventions for an Ebola epidemic of this scale has not yet been studied. Here, we describe the relationship between various media reported events – including interventions – and changes in epidemic behavior between April 14 and October 11, 2014 in Sierra Leone and Liberia.

Using WHO aggregate reporting data,^2^^,^ 3 we estimated observed reproductive number (*R*_*Obs*_) over time for Sierra Leone and Liberia using the Incidence Decay and Exponential Adjustment (IDEA) method.^4^ This was achieved by optimizing both *R_0_* and *d* over 10 serial intervals using the GRG non-linear algorithm, where the objective function was set to minimize the sum of square differences between modeled cumulative incidence and actual cumulative incidence data from the WHO. We defined the observed reproductive number as follows:

\begin{equation*}R_{Obs} = \frac{R_{0}}{(1+d)^{t} } \end{equation*}

Which describes the *observed* number of secondary infections per infected individual for a given serial interval (*t*), defined as an integer value equal to the disease incubation period plus one half of the illness duration.

Using HealthMap (healthmap.org), we identified news stories of disease control interventions and “aggravating events” – namely, events that were likely to cause an increase in contact rate and thus, *R_Obs_* (e.g. widely-publicized traditional burials). Additional detail regarding the nature of these events, including chronology, is available in the Appendix. For clarity, we classified reported interventions into one of three categories: providing education, minimizing contact, and strengthening healthcare. Aggravating events were not further classified. Using a serial interval of 18 days, we then charted *R_Obs_*, reported interventions, and reported aggravating events over time. In order to assess the relationship between media reports of interventions and transmission, we assumed that contact rate and *R_Obs_* would remain constant for the time frame considered in the absence of control measures or aggravating events.^5^

**Relationship between media reports of interventions and aggravating events on observed reproductive number in Sierra Leone and Liberia.**
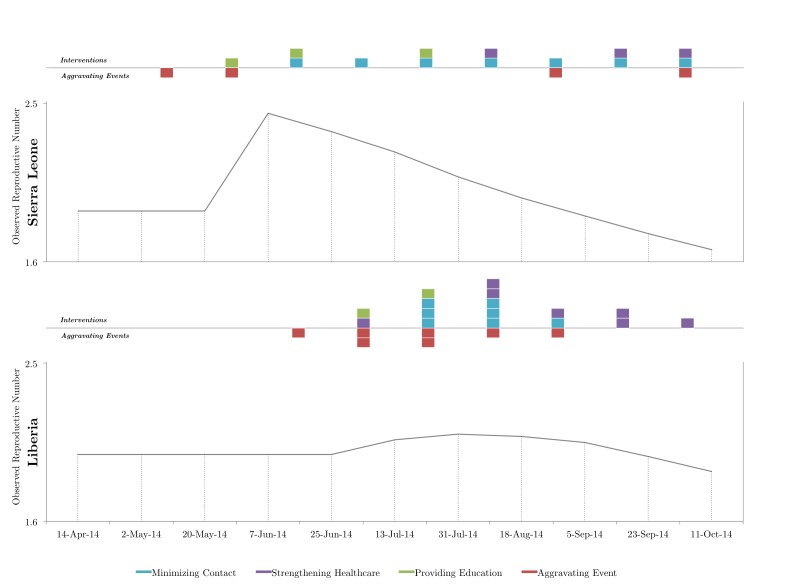
All interventions and aggravating events are scale-less (e.g. 1 event is denoted with 1 block) and plotted by time of occurrence (i.e. serial interval of occurrence).

**The *R_Obs_* curves differed considerably between the two countries (Figure). From inspection, media reports of aggravating events generally preceded a temporary increase in *R_Obs_*; however, this effect was occasionally dampened in the presence of reported control measures. Nevertheless, we found that the number of aggravating event reports in a given serial interval was positively correlated with the change in *R_Obs_*, as seen from the vantage point of the following serial interval (Liberia, *r^2^* = .96 and Sierra Leone, *r^2^* = .52). Meanwhile, media reports of interventions that did not coincide with reported aggravating events were typically followed by a sustained decrease in *R_Obs_*.

Media reports of control measures may have been followed by a lasting reduction in transmission because such measures were typically implemented at the national scale (e.g. aid distribution). Conversely, media reports of aggravating events may have preceded transient, country-level increases in transmission because such events generally localized in nature (e.g. nurses fleeing clinics).

This qualitative analysis indicates local aggravating events and regional interventions, as reported in real-time by media outlets, track changes in observed reproductive number. In the future, media reported events – acting as proxies for qualitative changes in epidemic behavior – could potentially be incorporated into epidemic modeling efforts to improve mid-outbreak case projections.

## Competing Interest

The authors declare no competing interests.
